# Genetic diversity of Indonesian cattle breeds based on microsatellite markers

**DOI:** 10.5713/ajas.18.0283

**Published:** 2018-08-27

**Authors:** Paskah Partogi Agung, Ferdy Saputra, Moch Syamsul Arifin Zein, Ari Sulistyo Wulandari, Widya Pintaka Bayu Putra, Syahruddin Said, Jakaria Jakaria

**Affiliations:** 1 Research Center for Biotechnology-Indonesian Institute of Sciences, Cibinong 16911, West Java, Indonesia; 2 Laboratory of Genetics Indonesia, Cikarang Technopark, Bekasi, West Java 17550, Indonesia; 3 Research Center for Biology-Indonesian Institute of Sciences, Cibinong 16911, West Java, Indonesia; 4 Faculty of Animal Science, Bogor Agricultural University, Darmaga Campus, Bogor 16680, Indonesia

**Keywords:** Genetic Diversity, Indonesian, Cattle Breed, Microsatellite

## Abstract

**Objective:**

This research was conducted to study the genetic diversity in several Indonesian cattle breeds using microsatellite markers to classify the Indonesian cattle breeds.

**Methods:**

A total of 229 DNA samples from of 10 cattle breeds were used in this study. The polymerase chain reaction process was conducted using 12 labeled primers. The size of allele was generated using the multiplex DNA fragment analysis. The POPGEN and CERVUS programs were used to obtain the observed number of alleles, effective number of alleles, observed heterozygosity value, expected heterozygosity value, allele frequency, genetic differentiation, the global heterozygote deficit among breeds, and the heterozygote deficit within the breed, gene flow, Hardy-Weinberg equilibrium, and polymorphism information content values. The MEGA program was used to generate a dendrogram that illustrates the relationship among cattle population. Bayesian clustering assignments were analyzed using STRUCTURE program. The GENETIX program was used to perform the correspondence factorial analysis (CFA). The GENALEX program was used to perform the principal coordinates analysis (PCoA) and analysis of molecular variance. The principal component analysis (PCA) was performed using adegenet package of R program.

**Results:**

A total of 862 alleles were detected in this study. The INRA23 allele 205 is a specific allele candidate for the Sumba Ongole cattle, while the allele 219 is a specific allele candidate for Ongole Grade. This study revealed a very close genetic relationship between the Ongole Grade and Sumba Ongole cattle and between the Madura and Pasundan cattle. The results from the CFA, PCoA, and PCA analysis in this study provide scientific evidence regarding the genetic relationship between Banteng and Bali cattle. According to the genetic relationship, the Pesisir cattle were classified as *Bos indicus* cattle.

**Conclusion:**

All identified alleles in this study were able to classify the cattle population into three clusters i.e. *Bos taurus* cluster (Simmental Purebred, Simmental Crossbred, and Holstein Friesian cattle); *Bos indicus* cluster (Sumba Ongole, Ongole Grade, Madura, Pasundan, and Pesisir cattle); and *Bos javanicus* cluster (Banteng and Bali cattle).

## INTRODUCTION

Indonesia has many native breeds of cattle, including Bali cattle, Pesisir, Sumba Ongole, Madura, Aceh, Grati, Ongole Grade, Katingan, Sumbawa, Pasundan, Jabres, and Galekan (the characteristics were described in Sutarno and Setyawan [1] and MARI [2]). Due to their vital role in the Indonesian socio-economy, conservation and breeding programs for Indonesian cattle breeds should be well-designed based on their potency and genetic information. However, several local breeds of cattle in Indonesia remain unrecorded, and there is a lack of scientific information regarding them as well. This condition leads to the *in-situ* conservation or breeding program becoming ineffective. The Pasundan cattle are an example of unrecorded local breed cattle in Indonesia [3].

Information about genetic diversity is needed to perform a conservation program especially regarding Indonesian local cattle resources and to provide an opportunity for farmers to develop animal breeding businesses. The development of molecular genetics analysis has made it possible to study the potency of certain cattle breeds at the deoxyribonucleic acid (DNA) level. Microsatellites are almost ideal genetic markers because they are abundant, codominant, highly polymorphic, and spread out across the entire euchromatic part of the genome [4].

The microsatellites markers can be used for estimating the genetic distance [5], the relationship among livestock breeds [6], paternity testing [7], and the genetic diversity [8]. This research was conducted to study the genetic diversity in several Indonesian cattle breeds using microsatellite markers as scientific evidence to classify the Indonesian cattle breeds that can be used to design breeding and conservation strategy for the Indonesian cattle breeds in future.

## MATERIALS AND METHODS

### Blood sample and DNA collection

This study was conducted following the guidelines of research implementation included in the Indonesian Institute of Sciences Regulation number 08/e/2013 about the ethical clearance of research and scientific publication. Ethical Clearance Committee of the Indonesian Institute of Sciences, Jakarta, Indonesia has approved all procedures related to the used of animal in this study (Register No. 9879/WK/HK/XI/2015). A total of 229 head of cattle including Simmental Purebred (n = 19), Simmental Crossbred (n = 27), Ongole Grade (n = 27), Bali (n = 20), Pesisir (n = 13), Holstein Friesian (n = 20), Sumba Ongole (n = 38), Madura (n = 20), Banteng (n = 20), and Pasundan cattle (n = 25) were used for blood sampling. Blood samples (3 to 5 mL) were taken from the *coccygeal* vein using *Venoject* and collected in *Vaccutainer* tubes containing an anticoagulant. The blood samples were used in the DNA extraction process using the DNeasy Blood & Tissue Kit (Qiagen, Hilden, Germany) following the producer’s method.

### Primer and amplification

A total of 12 microsatellite labeled primers with high polymorphism information content (PIC) value (part of the 30 primers recommended by Food and agriculture organization of the United Nations [FAO]) were used in the polymerase chain reaction (PCR) process (primers sequence, annealing temperature, range of PCR product size, and label used were based on Agung [9]). The PCR reagent composition was as follows: KAPA2G Robust Hot Start Ready Mix PCR Kit (Kapa Biosystems, Cape Town, South Africa) (18 μL), forward and reverse labeled primers (200 ng/μL), nuclease free water, and DNA samples (5 to 30 ng/μL). The program in the PCR machine (Eppendorf, Hamburg, Germany) was set at 94°C; 5 min (1 cycle), 35 cycles consisting of three stages: i) 94°C; 30 s, ii) 51°C to 59°C; 30 s (depending on primers), and iii) 72°C; 30 s, followed by one cycle at 72°C; 5 min. The PCR products were then visualized by electrophoresis using 2% agarose gel and followed by SyBr staining and captured in GBOX documentation System (Syngene, Cambridge, UK). Multiplex DNA fragment analysis was conducted afterwards for allele identification in 1st BASE Laboratory, Malaysia.

### Data analysis

Data of allele’s size (unit in base pairs) were generated using the multiplex DNA fragment analysis. The data was processed using CONVERT version 1.3.1 [10] to convert the size of alleles observed for each individual sample to assure suitability for further data analysis. The converted data was processed using POPGEN version 1:32 program [11] to generate the observed number of alleles (n_a_), effective number of alleles (n_e_), observed heterozygosity value (H_o_), expected heterozygosity value (H_e_), genetic differentiation (F_ST_), the global heterozygote deficit among breeds (F_IT_), and the heterozygote deficit within the breed (F_IS_), gene flow (Nm), Hardy-Weinberg equilibrium (HW), and allele frequency. The converted data was also processed using CERVUS version 3.0.7 program [12] to obtain the PIC value. The genetic distance value was used to make a dendrogram to illustrate the relationship among cattle populations using MEGA version 6.0 [13]. Bayesian clustering assignments were analyzed using STRUCTURE version 2.2 [14]. Ten independent runs were performed for each K between 2 and 10, with a burn-in period of 1,000,000 iterations followed by 1,000,000 iterations of the Markov Chain Monte Carlo algorithm. The STRUCTURE HARVESTER [15], which implements the Evanno method [16] was used to identify the optimal groups (K). The correspondence factorial analysis (CFA), principal coordinates analysis (PCoA), and principal component analysis (PCA) were conducted to determine the relationship among breeds. The GENETIX program [17] was used to perform the CFA and the GENALEX version 6.1 program [18] was used to perform the PCoA and analysis of molecular variance (AMOVA). The PCA were performed using adegenet package [19] of R version 3.2.0 (2015.4.16) [20]. The F-statistics were estimated based on Weir and Cockerham [21] and the genetic distance value in ten breeds populations were estimated based on Nei’s genetic identity and genetic distance [22].

## RESULTS

### Microsatellite polymorphism

Twelve microsatellite loci revealed high polymorphism to evaluate the genetic diversity. The 12 microsatellite loci from the entire population were analyzed and 862 alleles consisting of 86 alleles detected in the Simmental Purebred, 103 alleles in the Simmental Crossbred, 118 alleles in the Ongole Grade, 60 alleles in the Bali, 70 alleles in the Pesisir, 78 alleles in the Holstein Friesian, 98 alleles in the Sumba Ongole, 100 alleles in the Madura, 56 alleles in the Banteng, and 93 alleles in the Pasundan cattle.

The observed heterozygosity value ranged from 0.417±0.321 (Banteng) to 0.719±0.158 (Simmental Crossbred) and the expected heterozygosity value ranged from 0.550±0.248 (Banteng) to 0.796±0.151 (Madura). The expected heterozygosity was higher than the observed heterozygosity in all populations studied (Table 1). F-statistics were estimated in a fixation index as genetic differentiation (F_ST_), the global heterozygote deficit among ten cattle breeds (F_IT_), and the heterozygote deficit within the breed (F_IS_) among the twelve microsatellite markers (Table 2).

Among the 12 microsatellite markers, the estimation of the fixation index has been determined for F_ST_, F_IT_, and F_IS_ with values ranging from 0.140 to 0.538, 0.055 to 0.522, and −0.099 to 0.377, respectively. The estimated mean values of the total inbreeding (F_IT_), within line inbreeding (F_IS_) and genetic distance were 0.367, 0.160, and 0.243 respectively. The PIC values ranged from 0.715 to 0.935. Therefore, the microsatellite markers in this study were very informative.

The result of genetic distance analysis shows that the Simmental Purebred and the Simmental Crossbred subpopulation have the lowest genetic distance value. In contrast, the Simmental Purebred and the Banteng subpopulation have the highest genetic distance value (Table 3). The locus INRA23 allele 205 is a specific allele candidate for the Sumba Ongole cattle, while the allele 219 is a specific allele candidate for Ongole Grade. Others specific breed allele candidates in this study are shown in Table 4, however, the specific allele candidates still need to be validated by further studies.

### Genetic distance and structure of breeds

**Figure 1:**
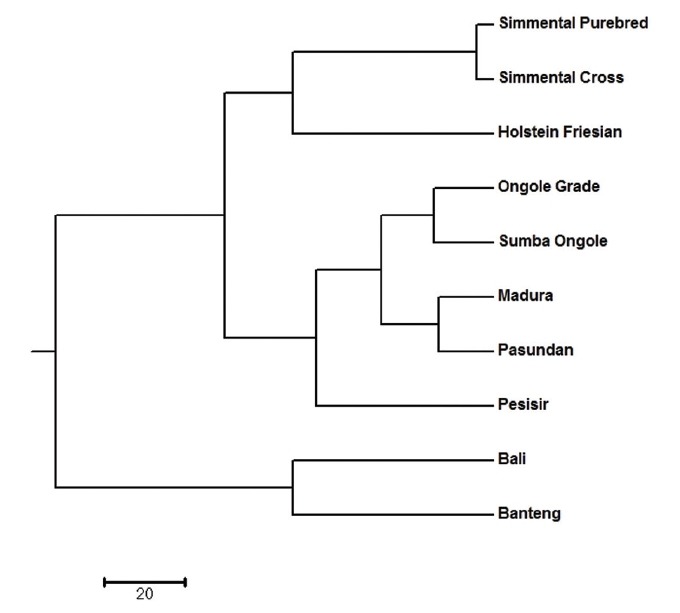
Dendrogram of the Indonesian cattle breeds population using UPGMA method based on Nei [22].

All identified alleles in this study could be used to classify the cattle population into clusters. There are subpopulations that are closely related, and they form their own respective cluster. The dendrogram of unweighted pair-group method with arithmetic mean (UPGMA) revealed three main clusters, with Simmental Purebred, Simmental Crossbred, and Holstein Friesian breeds in the first cluster (*Bos taurus* cluster); Ongole Grade, Sumba Ongole, Madura, Pasundan, and Pesisir breeds in the second cluster (*Bos indicus* cluster); Bali and Banteng breeds in the third cluster (*Bos javanicus* cluster) (Figure 1). The clustering of breeds into three groups by UPGMA analysis highlights the presence of clear genetic separation between breeds in different groups.

**Figure 2:**
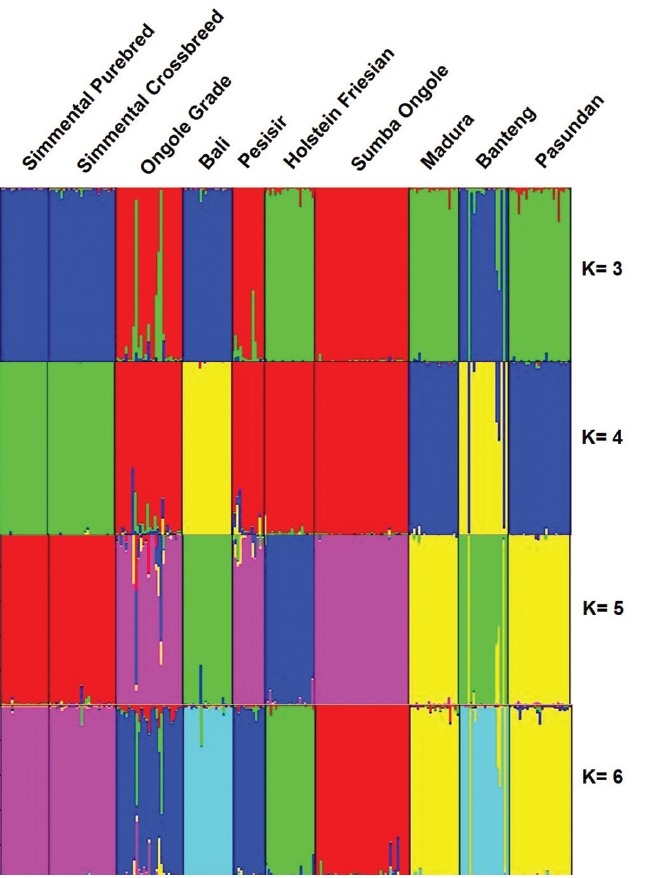
Genetic structures of the Indonesian cattle breeds. Black lines separate individual populations whose names were indicated.

Using STRUCTURE HARVESTER, K optimal was obtained at K = 5. At K = 5, Madura and Pasundan cattle have genetic similarities. Furthermore, Simmental Purebred and Simmental Crossbred cattle also have genetic similarities. In addition, the Holstein Friesian cattle were identified as a separated cluster. The STRUCTURE analysis generated quite similar interpretation (Figure 2) to the dendrogram of UPGMA.

**Figure 3:**
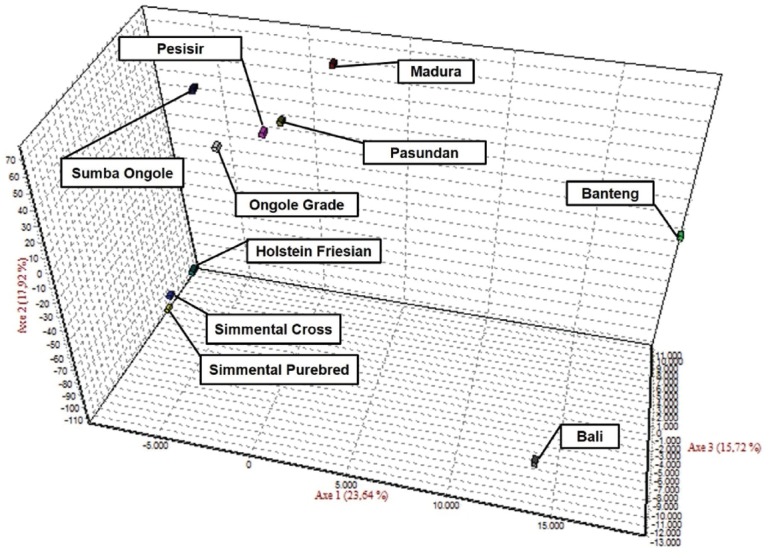
Correspondence factor analysis (CFA) of the Indonesian cattle breeds.

**Figure 4:**
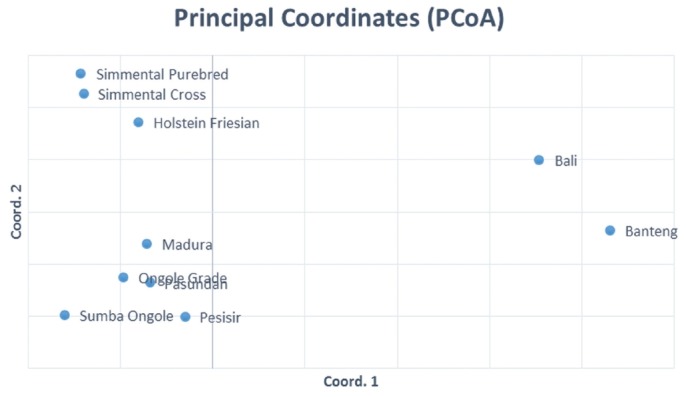
Principal coordinates analysis (PCoA) of the Indonesian cattle breeds.

**Figure 5:**
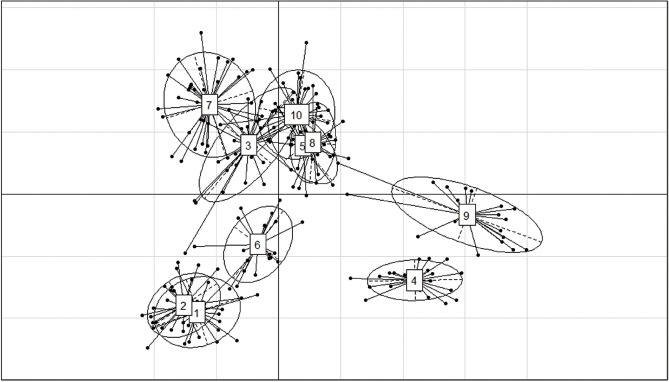
First and second components of a principle components analysis of 12 microsatellite loci genotypes from Simmental Purebred (1), Simmental Crossbred (2), Ongole Grade (3), Bali (4), Pesisir (5), Holstein Friesian (6), Sumba Ongole (7), Madura (8), Banteng (9), and Pasundan (10) population.

The results of the CFA (Figure 3), PCoA (Figure 4) and PCA (Figure 5) analysis also revealed three clusters: *Bos taurus*, *Bos indicus*, and* Bos javanicus*. Ongole Grade, Sumba Ongole, Madura, Pasundan, and Pesisir cattle were included in the *Bos indicus* cluster. Meanwhile, Simmental Purebred, Simmental Crossbred, and Holstein Friesian were clustered in *Bos taurus* cluster. Bali cattle and Banteng were separated in *Bos javanicus* cluster. The AMOVA result revealed that variation among individuals more varied (69%) than the variation in the inter-population (31%) (Table 5).

## DISCUSSION

### Microsatellite markers polymorphism

Utilization of five microsatellite markers (TGLA227, ETH225, BM1824, INRA005, and MM12) to evaluate the genetic diversity of Indonesian cattle breeds (Ongole Grade, Aceh, Bali, and Madura cattle) was reported and provides a phylogenetic relationships between the breeds [23]. However, the genetic diversity study based on microsatellite marker analysis will generate more reliable data when the number of microsatellite markers are increased [24]. This study is the first to report the genetic diversity in Indonesian cattle breeds based on 12 of the 30 microsatellite primers recommended by FAO. The expected heterozygosity value was higher than the observed heterozygosity value in all populations studied. This condition can be explained by several factors, including null alleles, assortative mating, the Wahlund effect, selection against heterozygotes, inbreeding, or a combination of all these factors [25]. In addition, the low value of heterozygosity indicates that certain breeds are relatively well-conserved [26]. The informative locus in genetic application should have PIC value more than 0.5 and H_e_>0.6 [27]. In addition, the estimation of genotypic diversity in heterozygosity and PIC value informativeness of microsatellite markers were used to determine animal breed selection [28]. In this study, 12 microsatellite markers selected were highly informative for the ten cattle breeds and are appropriate for discrimination as well. The fixation indices (F_IS_, F_IT_, and F_ST_) among ten cattle breeds in this study were positive (0.160, 0.367, and 0.243) and indicates that there has been a selection process in the population.

Based on Agung et al [29], the morphological characteristics between the Simmental Purebred and the Simmental Crossbred were significantly different. The results of this study were in accordance with previous study [30] that used the 12 microsatellite markers to separate the Simmental Purebred from the Simmental Crossbred populations and reported that the TGLA53 (allele 168) was the candidate for specific breed allele in Simmental Purebred while the SPS115 (allele 250) and TGLA122 (allele 181) were candidates for specific breed alleles in Simmental Crossbred. While for the Simmental population in this study, only SPS115 allele 250 was observed. The INRA23 allele 205 is a specific allele candidate for the Sumba Ongole cattle, while the allele 219 is a specific allele candidate for Ongole Grade although the allele frequencies (0.0143 and 0.0185 respectively) of these alleles were low. However, the INRA23 (allele 205 and 219) can be proposed as specific allele to separate these two Ongole breeds.

### Diversity of Indonesian cattle breeds

In general, the Ongole Grade cattle are a crossbred of uncontrolled mating of Java cattle and Sumba Ongole cattle [31]. This study revealed a very close genetic relationship between the Ongole Grade and Sumba Ongole cattle. Hence, the scientific evidence of the origin of Ongole Grade cattle in Indonesia based on microsatellite markers in this study can be presented as well.

Genetic distances based on PCA can illustrate the relative effect of intra and interspecies variation [32]. In addition, the PCA analysis results cluster individuals only based on their genotypes; hence, no assumptions can be made regarding the HW or linkage equilibrium as well [33]. The PCA analysis indicated a close relationship between the Simmental Crossbred population and the Simmental Purebred population. The Madura, Pasundan, and Pesisir cattle population are closely related as well. The value of the genetic distance can be influenced by many factors, including the number of the population used in the study and the objectives of the breeding [34], as well as the massive introgression of possible breeds of cattle because of their geographical relationship [35].

High genetic similarities between Banteng and Bali cattle in this study are reasonable because the Bali cattle are the result of a direct domestication of wild Banteng in the Bali Island or in Blambangan (East Java Province) and proposed as the most suitable cattle breed for sustainable small farming in Indonesia [36]. The results from the CFA, PCoA and also PCA analysis in this study confirmed the previous report regarding the genetic relationship between Banteng and Bali cattle. Based on mitochondrial DNA and 16 microsatellite markers analysis, Bali cattle are closely related to Banteng. In addition, Bali, Madura and Pesisir cattle were distinct from Sahiwal, Red Sindhi, Tharparkar, Hariana and Nellore cattle [37]. Since 1967, the Indonesian Government appointed the Sapudi Island as the center of Madura cattle pure breeding program. Hence, the cross-breeding program for Madura cattle is prohibited under any circumstances on Sapudi Island. However, the great majority of the conventional Madura cattle will be crossbreds [38]. Based on Sutarno et al [23], the Madura cattle were clearly distinct from PO cattle, Aceh cattle, and Bali cattle. In contrast, based on CFA analysis in this study, the Madura cattle were clustered in the *Bos indicus* cluster (Sumba Ongole, Ongole Grade, Pesisir, and Pasundan cattle) (Figure 3). This condition can be caused by introgression of the zebu cattle was more intensive in the Madura cattle breeding program.

Pasundan cattle have been classified as newest Indonesian local cattle based on the Decree of the Minister of Agriculture number 1051/Kpts/SR.120/10/2014. These cattle are often called *kacang* (bean) cattle because of their relatively small body size [39], and they are produced from the crossing program between *Bos javanicus* and *Bos indicus*. The result of the genetic distance analysis shows that the Madura and the Pasundan cattle have low genetic distance value; thus, it can be interpreted that these cattle breeds have a very close genetic relationship, and this can serve as scientific evidence on the origin of the Pasundan cattle.

Pesisir cattle have been classified as one of Indonesian cattle breeds based on the Decree of the Minister of Agriculture Number 2908/Kpts/OT.140/6/2011. The Pesisir cattle have a unique performance due to their small body (having the smallest size among other Indonesian cattle breeds), and their natural habitat was only in West Sumatera, Indonesia [1]. According to the genetic relationship, the Pesisir cattle are clustered together with Ongole Grade, Sumba Ongole, Madura, and Pasundan cattle in *Bos indicus* cluster. This result can also be scientific evidence that the Pesisir cattle in West Sumatera, Indonesia are a type of *Bos indicus* cattle breeds.

The AMOVA is an essential element of the molecular eco logist’s toolkit. Coupled with hierarchical permutation tests, the approach facilitates rigorous statistical inference about the distribution of genetic variation in natural populations [40]. The AMOVA result in this study revealed that variation among individuals more varied than the variation in the inter-population. This condition can be caused by the sample populations used in this study (based on microsatellite markers of 10 population), which are mostly native or local Indonesian cattle breeds. In addition, designing breeding programs of Indonesian cattle breeds is very important to prevent genetic diversity loses.

## CONCLUSION

The 12 microsatellite markers in this study are highly polymorphic and highly informative in detecting the level of genetic diversity among the Indonesian cattle breeds. All identified alleles in this study were able to classify the cattle population into three clusters i.e. *Bos taurus* cluster (Simmental Purebred, Simmental Crossbred, and Holstein Friesian cattle); *Bos indicus* cluster (Sumba Ongole, Ongole Grade, Madura, Pasundan, and Pesisir cattle); and *Bos javanicus* cluster (Banteng and Bali cattle).

## Figures and Tables

**Table 1:** Summary statistic of the mean number of observed allele (N_a_), mean number of effective alleles (N_e_), observed (H_o_), and expected (H_e_) heterozygosities, and PIC observed in the ten breeds

Breed1)	N	N_a_±SD	N_e_±SD	H_o_±SD	H_e_±SD	PIC
A	19	7.167±2.250	4.005±1.392	0.615±0.171	0.745±0.092	0.687
B	27	8.583±2.875	4.915±2.006	0.719±0.158	0.761±0.158	0.722
C	27	9.833±2.406	5.067±2.075	0.651±0.177	0.788±0.085	0.749
D	20	5.455±2.423	3.361±1.320	0.632±0.261	0.648±0.236	0.542
E	13	6.364±1.859	3.590±1.384	0.566±0.271	0.704±0.147	0.589
F	20	7.091±2.212	3.767±1.294	0.581±0.144	0.727±0.094	0.611
G	38	8.167±2.038	3.355±1.353	0.553±0.258	0.662±0.148	0.619
H	20	8.417±2.843	5.283±2.114	0.587±0.232	0.796±0.151	0.738
I	20	4.750±1.815	2.633±1.185	0.417±0.321	0.550±0.248	0.495
J	25	7.750±2.927	4.705±2.059	0.501±0.240	0.740±0.212	0.692

PIC, polymorphism information content; SD, standard deviation.

1) A, Simmental Purebred; B, Simmental Crossbred; C, Ongole Grade; D, Bali; E, Pesisir; F, Holstein Friesian; G, Sumba Ongole; H, Madura; I, Banteng; J, Pasundan.

**Table 2:** Fixation indices (F_IS_, F_IT_, and F_ST_) among ten cattle breeds

Loci	N_a_	H_o_	H_e_	PIC	F_IS_	F_IT_	F_ST_	Nm	HW
BM1824	23	0.464	0.862	0.847	0.324	0.474	0.222	0.874	***
ILST6	25	0.442	0.881	0.869	0.377	0.516	0.223	0.869	***
TGLA126	23	0.626	0.934	0.928	0.186	0.342	0.191	1.057	NS
TGLA53	32	0.633	0.893	0.885	0.158	0.302	0.172	1.207	***
TGLA227	32	0.461	0.917	0.909	0.214	0.502	0.367	0.431	***
TGLA122	30	0.698	0.927	0.920	0.121	0.267	0.166	1.256	NS
ETH225	31	0.731	0.940	0.935	0.035	0.217	0.188	1.077	NS
INRA23	24	0.632	0.911	0.903	0.119	0.341	0.252	0.743	***
SPS113	27	0.890	0.921	0.914	−0.099	0.055	0.140	1.536	NS
SPS115	19	0.550	0.825	0.810	0.207	0.395	0.237	0.806	***
BM1818	14	0.404	0.793	0.768	0.316	0.467	0.220	0.886	***
CSSM66	26	0.591	0.727	0.715	−0.034	0.522	0.538	0.215	***
Mean					0.160	0.367	0.243	0.913	
SD					0.042	0.041	0.032	0.103	

N_a_, number of allele; H_o_, observed heterozygosity; H_e_, expected heterozygosity; PIC, polymorphism information content; Nm, gene flow; HW, Hardy-Weinberg equilibrium; NS, not significant; SD, standard deviation.

*** p<0.001.

**Table 3:** Genetic distance value in ten breeds populations based on Nei’s genetic identity (above diagonal) and genetic distance (below diagonal)

Population	A	B	C	D	E	F	G	H	I	J
A	-	0.9240	0.3817	0.1262	0.1200	0.3478	0.3313	0.2284	0.0123	0.2498
B	0.0790	-	0.4428	0.1350	0.1412	0.3957	0.4023	0.2484	0.0216	0.2717
C	0.9632	0.8146	-	0.2012	0.5449	0.4143	0.7467	0.4798	0.1412	0.7061
D	2.0698	2.0027	1.6032	-	0.1990	0.2204	0.1179	0.1351	0.3708	0.1207
E	2.1204	1.9575	0.6072	1.6147	-	0.3094	0.4185	0.3015	0.1275	0.4406
F	1.0561	0.9272	0.8811	1.5125	1.1732	-	0.3913	0.1727	0.0449	0.1747
G	1.1047	0.9107	0.2922	2.1379	0.8710	0.9382	-	0.5551	0.1290	0.5857
H	1.4766	1.3928	0.7344	2.0018	1.1991	1.7564	0.5885	-	0.4126	0.7649
I	4.3995	3.8366	1.9575	0.9921	2.0597	3.1032	2.0477	0.8852	-	0.2657
J	1.3869	1.3029	0.3480	2.1141	0.8195	1.7449	0.5349	0.2680	1.3253	-

A, Simmental Purebred; B, Simmental Crossbred; C, Ongole Grade; D, Bali; E, Pesisir; F, Holstein Friesian; G, Sumba Ongole; H, Madura; I, Banteng; J, Pasundan.

**Table 4:** Summary of specific alleles candidate in the cattle breeds studied

Markers	A	B	C	D	E	F	G	H	I	J
BM1824	-	-	-	190	-	-	197	161	216	193
	-	-	-	194	-	-	199	167	-	-
	-	-	-	196	-	-	-	187	-	-
ILST6	-	-	276	282	301	-	300	267	285	289
	-	-	284	-	303	-	-	307	-	-
TGLA126	-	-	103	113	-	-	-	-	-	-
	-	-	129	-	-	-	-	-	-	-
TGLA53	164	132	159	159	151	139	-	-	-	-
	168	150	165	-	-	141	-	-	-	-
	-	154	-	-	-	145	-	-	-	-
	-	156	-	-	-	-	-	-	-	-
	-	162	-	-	-	-	-	-	-	-
TGLA227	95	-	87	-	-	69	84	68	-	75
	-	-	91	-	-	87	86	72	-	103
	-	-	-	-	-	103	88	74	-	-
	-	-	-	-	-	105	94	98	-	-
	-	-	-	-	-	-	96	-	-	-
	-	-	-	-	-	-	100	-	-	-
TGLA122	157	135	134	166	-	106	-	-	-	156
	171	165	165	-	-	186	-	-	-	-
	185	-	-	-	-	-	-	-	-	-
ETH225	-	-	128	-	163	-	-	133	130	148
	-	-	-	-	-	-	-	137	160	-
	-	-	-	-	-	-	-	151	-	-
	-	-	-	-	-	-	-	157	-	-
INRA23	-	-	219	-	-	-	205	-	-	-
SPS113	-	153	-	140	-	-	-	123	122	-
	-	-	-	146	-	-	-	125	128	-
	-	-	-	-	-	-	-	127	136	-
	-	-	-	-	-	-	-	159	-	-
SPS115	264	-	-	-	-	-	258	-	-	241
	-	-	-	-	-	-	262	-	-	249
	-	-	-	-	-	-	264	-	-	253
	-	-	-	-	-	-	-	-	-	255
BM1818	-	-	248	-	280	-	-	272	278	282
CSSM66	197	177	192	-	-	-	194	170	210	-
	-	-	-	-	-	-	-	224	216	-
	-	-	-	-	-	-	-	228	220	-

A, Simmental Purebred; B, Simmental Crossbred; C, Ongole Grade; D, Bali; E, Pesisir; F, Holstein Friesian; G, Sumba Ongole; H, Madura; I, Banteng; J, Pasundan.

**Table 5:** Analysis of molecular variance among ten cattle breeds

Source	df	SS	MS	Est. Var	%
Among population	9	1,056.921	117.436	4.707	31
Among individual	219	2,318.900	10.589	10.589	69
Total	228	3,375.821	-	15.296	100

df, degrees of freedom; SS, sum of squared; MS, mean of squared; Est.Var., estimated variance.
